# Barriers to dog rabies vaccination during an urban rabies outbreak: Qualitative findings from Arequipa, Peru

**DOI:** 10.1371/journal.pntd.0005460

**Published:** 2017-03-17

**Authors:** Ricardo Castillo-Neyra, Joanna Brown, Katty Borrini, Claudia Arevalo, Michael Z. Levy, Alison Buttenheim, Gabrielle C. Hunter, Victor Becerra, Jere Behrman, Valerie A. Paz-Soldan

**Affiliations:** 1 Department of Biostatistics and Epidemiology, University of Pennsylvania Perelman School of Medicine, Philadelphia, Pennsylvania, United States of America; 2 Zoonotic Disease Research Lab, Universidad Peruana Cayetano Heredia, Lima, Perú; 3 Department of Family and Community Health, University of Pennsylvania School of Nursing, Philadelphia, Pennsylvania, United States; 4 Center for Communication Programs, Johns Hopkins Bloomberg School of Public Health, Baltimore, Maryland, United States; 5 Microred Mariano Melgar, Ministerio de Salud, Arequipa, Perú; 6 Department of Economics, University of Pennsylvania School of Arts and Sciences, Philadelphia, Pennsylvania, United States of America; 7 Department of Sociology, University of Pennsylvania School of Arts and Sciences, Philadelphia, Pennsylvania, United States of America; 8 Department of Global Community Health and Behavioral Sciences, Tulane University School of Public Health and Tropical Medicine, New Orleans, Louisiana, United States; 9 Facultad de Salud Pública y Administración, Universidad Peruana Cayetano Heredia, Lima, Perú; Centers for Disease Control and Prevention, UNITED STATES

## Abstract

**Background:**

Canine rabies was reintroduced to the city of Arequipa, Peru in March 2015. The Ministry of Health has conducted a series of mass dog vaccination campaigns to contain the outbreak, but canine rabies virus transmission continues in Arequipa’s complex urban environment, putting the city’s 1 million inhabitants at risk of infection. The proximate driver of canine rabies in Arequipa is low dog vaccination coverage. Our objectives were to qualitatively assess barriers to and facilitators of rabies vaccination during mass campaigns, and to explore strategies to increase participation in future efforts.

**Methodology/Principal findings:**

We conducted 8 focus groups (FG) in urban and peri-urban communities of Mariano Melgar district; each FG included both sexes, and campaign participants and non-participants. All FG were transcribed and then coded independently by two coders. Results were summarized using the Social Ecological Model. At the individual level, participants described not knowing enough about rabies and vaccination campaigns, mistrusting the campaign, and being unable to handle their dogs, particularly in peri-urban vs. urban areas. At the interpersonal level, we detected some social pressure to vaccinate dogs, as well as some disparaging of those who invest time and money in pet dogs. At the organizational level, participants found the campaign information to be insufficient and ill-timed, and campaign locations and personnel inadequate. At the community level, the influence of landscape and topography on accessibility to vaccination points was reported differently between participants from the urban and peri-urban areas. Poor security and impermanent housing materials in the peri-urban areas also drives higher prevalence of guard dog ownership for home protection; these dogs usually roam freely on the streets and are more difficult to handle and bring to the vaccination points.

**Conclusions:**

A well-designed communication campaign could improve knowledge about canine rabies. Timely messages on where and when vaccination is occurring could increase dog owners’ perception of their own ability to bring their dogs to the vaccination points and be part of the campaign. Small changes in the implementation of the campaign at the vaccination points could increase the public’s trust and motivation. Location of vaccination points should take into account landscape and community concerns.

## Author summary

Canine rabies was reintroduced in Arequipa, Peru in March 2015, a rare event in an area previously declared free of transmission. In Arequipa, annual mass dog vaccination is practiced as a preventive strategy, with additional campaigns being implemented since the recent detection of the virus. However, these additional efforts have not quelled the outbreak and low dog vaccination coverage is driving ongoing transmission. We conducted focus groups in urban and peri-urban areas of Arequipa to identify barriers to and facilitators of canine vaccination during mass campaigns. Based on our findings, communication campaigns should seek to increase knowledge about canine rabies and the vaccination campaign, and provide timely messages on where and when vaccination is occurring. Small changes at the campaign’s vaccination points could increase public’s trust. Finally, there are differences between urban and peri-urban areas, such as landscape and topography that affect participation in mass vaccination campaigns and that should be considered when selecting locations for vaccination points.

## Introduction

The city of Arequipa, Peru is in the midst of an urban rabies epidemic. The first rabid dog was detected in March 2015, a rare instance of canine rabies reintroduction into an area previously declared free of transmission [1]. To date no human cases have been detected in Arequipa; however, continued transmission in dog populations puts the almost one million inhabitants of the city at risk of infection. Annual mass dog vaccination campaigns were instrumental in eliminating the disease from Arequipa in the 1990s. Unfortunately, this achievement was followed by low vaccination coverage in the years preceding the current outbreak [2]. Following the reemergence of the rabies virus in the city of Arequipa, the Ministry of Health of Peru (MOH) initiated additional vaccination campaigns with varying frequency [3]. These efforts have failed to quell the epidemic in Arequipa’s complex urban environment. Particularly, the city’s landscape is characterized by the presence of large dry water channels crossing the city in which free-roaming owned and stray dogs move, mix, and breed.

Mass dog vaccination remains the most effective strategy to eliminate canine rabies and canine-mediated human rabies in developing countries [4–6]. In Latin America, a combination of intensive dog vaccination and surveillance efforts has produced dramatic decreases in canine and human cases [5]. However, in addition to reintroduction in Arequipa, canine rabies has been recently introduced to new areas in Latin America such as Jujuy and Salta in Argentina, Mato Grosso do Sul in Brazil and Loma Plata in Paraguay [1]. New efforts and strategies to control the disease in the region are critical.

Eliminating canine rabies is feasible; however, several attempts based on dog vaccination have failed to achieve adequate coverage in the Americas, Africa and Asia [5,7,8]. There are multiple potential drivers of low participation in vaccination campaigns in southern Peru and elsewhere. The social ecological model [9] is a theory-based model that recognizes the complexity of the socio-cultural system in which individuals make decisions and take actions. This theory emphasizes that individuals not only make decisions based on their own knowledge and experience (individual level factors), but are also influenced by their interpersonal relationships (e.g. norms, families and peers), organizations (e.g. health promotion and prevention activities of health services), their community (e.g. physical environment), and policies (e.g. national or state laws) [9].

At the individual level, the ability to restrain and handle dogs [10–15]; lack of time to attend vaccination campaigns [12,13,15]; lack of information [10,13–15]; and level of knowledge of rabies [14,16–20] have been shown to influence vaccination uptake. At the interpersonal level, social norms (i.e. those affecting dog ownership practices) and migration patterns can have a distinct impact on people seeking preventive services, such as vaccines for their dogs [21–25]. At the organizational level, location and number of rabies vaccination points may be particularly important [11,15,16]. Also, the quality and quantity of health messages about rabies and the rabies campaigns may impact people’s knowledge, but not necessarily their behaviors [26]. At the community level, distance and topography can act as barriers to providing and accessing health services in urban and peri-urban settings [27–30]; in rural settings in particular, distance has been reported as a barrier to achieving high dog rabies vaccination coverage [11,15,16,31]. At the policy level, efforts to eliminate canine rabies have been jeopardized by lack of funding and low political will [5,32].

In Peru, the MOH organizes annual mass canine rabies vaccination campaigns in all cities (affected and unaffected by canine rabies). Campaigns are held on either one or two weekend days, offer free vaccination in outdoor settings, and are voluntary. The MOH uses a cell-culture-based vaccine [33]. Campaign promotion is conducted at the local level, primarily via posters placed in health centers, corner stores, schools and other meeting places a couple of weeks or a few days before mass vaccination. The day before and the same day of mass vaccination, campaign staff promote the campaign on foot or from trucks using megaphones; their routes are somewhat haphazard. Locations of the vaccination points are determined a few days in advance; some locations are relatively permanent from year to year (e.g. the entrance to a health post), whereas others may be selected on the day of the campaign. Teams may also move during the course of the day from a lower-demand to a higher-demand location. Vaccination usually starts between 8:30 and 10:00 am, and ends between 1:00 and 3:00 pm. The dog population (coverage denominator) is estimated based on the human:dog ratio method. The true human:dog ratio is highly variable geographically [34] and the ratio used throughout Arequipa has been changing from 10:1 to 5:1 in the last two years making vaccination coverage estimation a fast moving target.

The reemergence of rabies in the city of Arequipa has been associated with low dog vaccination coverage [2] and a high density of free-roaming dogs (i.e. stray and owned dogs that spend unsupervised time in the streets and water channels) [2,3]. The social and physical aspects of urbanization in rapidly growing cities such as Arequipa may also facilitate the emergence of canine rabies and complicate its control [35–39]. Epidemics of rabies and other zoonotic pathogens are ongoing in major urban centers across Latin America and worldwide [5,40–46], and it is necessary to understand barriers to dog vaccination, as well as to assess people’s understanding of rabies transmission and prevention. The objective of this study was to qualitatively assess barriers to and facilitators of dog vaccination during the mass campaigns implemented by the MOH in Arequipa, Peru, as well as to explore strategies to increase participation in future campaigns.

## Methods

### Ethics statement

Institutional Review Board approval was obtained from Universidad Peruana Cayetano Heredia (approval identification number: 65369), Tulane University (approval identification number: 14–606720), and University of Pennsylvania (approval identification number: 823736).

### Study setting

**Fig 1:**
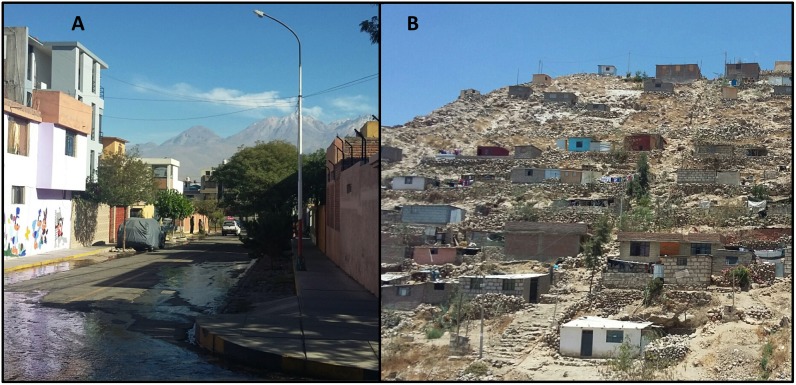
Urban (A) and peri-urban communities (B) where FG were conducted.

The study was conducted in the Mariano Melgar district (pop. 55,000) of the city of Arequipa, Peru’s second largest city. Arequipa is home to 969,000 people [47], and is situated at ~2,300 meters above sea level. The first detection of a rabid dog in the city of Arequipa occurred in March 2015 and by January 2016 (when our data were collected), 20 rabid dogs had been detected, of which 11 were found in Mariano Melgar. The city of Arequipa comprises communities spanning different stages of urbanization and different migration histories, from old established neighborhoods, to young neighborhoods, to recent invasions [48]. Within this gradient of development, young neighborhoods and recent invasions are often located on the periphery of the city (peri-urban area) and the older localities are nearer to the center (urban area) [38]. Compared to the urban area, peri-urban areas generally have lower socioeconomic status, fewer community resources, more security problems, and often more rugged and uneven terrain (Fig 1). As new neighborhoods mature into established neighborhoods with wealthier residents, homes are improved with better quality construction material and permanent utility connections, and connectivity with the rest of the city increases with better sidewalks, roads, and transportation access. One of Arequipa’s 14 districts, Mariano Melgar transects the city, running from the center to the periphery; the district continues to grow towards the outskirts of the city. In our study, participants represented either the urban or peri-urban areas of the city of Arequipa. The urban neighborhoods were founded several decades ago, while the peri-urban neighborhoods in our study originated around 2000 or later.

### Sampling strategy

Purposive sampling was used to select participants for a series of eight focus groups (FG). All participants were residents of urban and peri-urban neighborhoods and were recruited according to their geographic proximity to the site where the focus groups were held. To ensure that participants were not overly exposed to health promotion messages (i.e. posters about rabies vaccination campaign) or activities from a nearby health facility, all participants were recruited from homes located at least 6 blocks from the district's municipal building and health posts. The population in the Mariano Melgar district that lives 6 blocks or further from a health post or the municipal building was approximately 45,256 people. All participants in each focus group lived in the same neighborhood, but were recruited to represent a range of ages, gender, dog ownership (i.e. most participants had dogs, but in each FG we tried to have one person who did not currently own a dog), and dog vaccination status (i.e. we aimed to include both dog owners who had and had not participated in the vaccination campaigns in each FG).

### Recruitment approach

Research team members recruited participants door-to-door one day prior to the focus group. Pre-selecting blocks from a map, research assistants visited every third house within a block until they identified 2 or 3 individuals 18 years or older, preferably a dog owner, who were willing to participate. The same strategy was applied to all selected blocks until 15 individuals were invited to each focus group, to ensure a target attendance of 8–12 participants. The recruitment team explained the study goals and obtained written informed consent from all eligible participants. The recruitment team made follow-up visits the next day 45 minutes before the agreed-upon focus group time to remind participants. Consenting participants were picked up by car from their homes 10 to 15 minutes before the beginning of the FG and taken to the FG location. Participants were compensated for transportation home.

### Study population

Seventy individuals (18 to 81 years of age) participated in eight FG (4 groups each in the urban and peri-urban communities) over four days. The majority of participants were female (n = 54), but all focus groups had female and male participants (Table 1).

**Table 1:** Participant characteristics.

Study Area	FG	n	Age		Female %	Dog owner %	Vaccinated dog last year %			Place of birth %			Occupations
			median [range]				No dogs	Some dogs	All dogs	Urban Arequipa	Rural Arequipa	Other states	
Established community	1	9	47	[20–61]	88.9	100.0	0.0	44.4	55.6	--	--	--	Housewife, economist, student, small business owner
	2	8	48	[20–70]	87.5	87.5	14.3	28.6	57.1	--	--	--	Housewife, students, professor
	5	9	60	[40–81]	55.6	88.9	0.0	0.0	100.0	77.8	0.0	22.2	Housewife, farmer, personal coach, employee, nurse, retirees
	6	11	51	[18–72]	72.7	100.0	0.0	0.0	100.0	72.7	18.2	9.1	Housewife, lawyer, employees, students, small business owner
Young Community	3	8	25.5	[19–44]	75.0	87.5	0.0	42.9	57.1	25.0	12.5	62.5	Housewife, carpenter, seamstress/tailor, small business owner, driver
	4	7	46.5	[21–58]	71.4	100.0	0.0	33.3	66.7	33.3	33.3	33.3	Housewife, construction, student, small business owner
	7	11	35	[22–65]	91.0	91.0	30.0	30.0	40.0	45.5	9.1	45.5	Housewife, construction, daycare, seamstress/tailor, employee
	8	7	26	[18–52]	71.4	100.0	42.9	14.3	42.9	42.9	0.0	57.1	Housewife, driver
Total	--	70	43	[18–81]	77.1	94.2	10.9	23.4	65.6	51.9	11.5	36.5	--

### Data collection

FG guides were developed to cover four topics: dog ownership, dog ecology, and barriers to and facilitators of dog vaccination. Four facilitators conducted the FG: a social scientist experienced in qualitative research (VPS, PhD in public health), a research assistant (JB, BA in psychology), an infectious disease scientist (RCN, veterinarian and PhD in epidemiology, lead investigator of the study), and a note taker. All facilitators were Peruvian, and the infectious disease scientist has lived and/or worked in Arequipa for more than seven years.

### Data management and analysis

All FG discussions were digitally audio-recorded and transcribed, and detailed notes were taken throughout. An inductive coding process was used: we first grounded ourselves in the data to explore the topics that would emerge, not knowing what to expect [49]. Codes were then developed based on the emerging themes. Data were imported into ATLAS.ti [50] and coded by two members of the research team; any inconsistencies in the coding were discussed thoroughly until agreement, with codes added as needed. Transcripts that had been coded were recoded with the revised coding scheme. To address the main question of interest to the researchers, all data coded to themes related to canine rabies vaccination barriers and facilitators were summarized, stratified by urban vs. peri-urban communities, and younger (<30 years of age) and older participants.

## Results

Several barriers to and facilitators of dog rabies vaccination emerged from the FG, from logistical issues to those associated with the physical environment. We applied the social ecological model (SEM) as a framework to organize the vaccination barriers and facilitators at four levels: the individual level, interpersonal level, organizational level and the community level (Table 2). In this paper the community level refers to physical factors in the urban environment, consistent with the Centers for Disease Control and Prevention’s application of the model [51]. Our study did not explore the policy environment, the outermost level of the model.

**Table 2:** Summary of factors that influence canine rabies vaccination uptake.

Level	Main themes	Barriers Identified	Facilitators Identified
Individual	1.1 Insufficient knowledge about the vaccination campaign and rabies	• Lack of information about the campaign • Insufficient knowledge of rabies and rabies vaccination • Belief that purebred dogs are more vulnerable to illnesses than mongrels	• Exposure to communication about rabies and the vaccination campaign
	1.2 Mistrust in quality of vaccination services and vaccine	• Mistrust of vaccine quality and staff competence • Consider the service and attention not warm enough • Bad previous experiences at campaign	
	1.3 High perceived risk of rabies for dogs and families	• Attitude of indifference towards rabies and the campaign • Low motivation to invest the time or effort in vaccination	• Fear of rabies • Believe that dogs’ health guarantees family´s safety and health • Awareness of many canine rabies cases in their district
	1.4 Logistical factors	• Difficulty in transporting dogs to the vaccination sites • Risk of dog fights at vaccination sites • Low knowledge about the dog vaccination campaign	• Affection and sense of duty towards their dogs • Recent experience of dog bites
Interpersonal	2.1 Social norms regarding relationship with dogs and dog care	• Norms around dog ownership—functional relationship with the dog (e.g. guard dog) • No norm of walking dog on leashes	• Emotional relationship with the dog (e.g. pet)
	2.2 Social pressure from the community to vaccinate–or not	• Evasiveness/aggressiveness or mockery if asked about vaccinating dog • Perception that neighbors who do not vaccinate have more urgent needs	• Social pressure: not wanting to get in trouble if their dog bit another • Pressure to vaccinate from other people (e.g. family, veterinarians)
Organizational	3.1 Insufficient health promotion and communication for dog vaccination campaigns	• Untimely advertisement of time of campaigns • Difficulty understanding megaphone messages • Lack of advertisement of vaccination point locations • Insufficient identification of personnel	
	3.2 Inadequate location and low frequency of mass vaccination campaign	• Infrequent vaccination campaigns • Inadequate vaccination point locations.	• Door to door vaccination would help, particularly mentioned by peri-urban residents
	3.3 Limited personnel vaccinating during the campaign	• Long lines at vaccination points • Vaccination points accesible for short periods	• Gratuity of vaccine • Possibility of receiving other dog services at the same time, such as de-worming
Community	4.1 Distance to vaccination point and difficult topography	• Distance and access to vaccination campaigns • Steep slopes and unlevel terrain in peri-urban areas • Large avenues in urban areas	
	4.2 Local security and poor housing materials	• Poor housing material impedes keeping dogs within the house (i.e. they break loose). • Lack of animal care culture: dogs on the streets, finding their own food, not sterilized. This is worse in peri-urban area. • In peri-urban areas, where security is worse, people have multiple dogs–harder to manage in a vaccine campaign.	• Door to door campaign would facilitate process for those with multiple, possibly aggressive dogs–and dogs not used to having leashes

### Individual-level factors

#### Insufficient knowledge about the vaccination campaign and rabies in general

In all FG, participants reported a **lack of information about the campaign**: megaphone announcements may not be heard or are not clear, and there are too few promotional posters. Some found out about the vaccination campaign only when they saw it happening, and either rushed home to get their dogs or simply missed it. Others missed the campaign because they were at work or traveling, and had not learned of the campaign in advance. Finally, some participants recounted stories of people not visiting vaccination points because they thought vaccination would cost money.

There is also a **lack of information about rabies vaccination.** People were not sure when, how often, and how many times dogs should be vaccinated against rabies. One participant mentioned she had only vaccinated her dog once when it was young; several asked at what age dogs should start receiving vaccines, and another person stopped because his dog was old and he thought it no longer needed vaccines. A few participants believed dogs kept indoors do not need vaccines, as well as those who are either tame, never bite or are very small.

**Beliefs related to different dogs’ risks of illness** and “being replaceable” also seemed to affect owners’ vaccination behaviors. In all FG, participants stated that purebred dogs are more vulnerable to illnesses than mongrels. As such, purebreds are considered to be in greater danger of becoming infected at the campaigns by being exposed to other dogs that may carry pathogens or fleas. Mongrels were perceived as less likely to get ill, and also more easily replaceable if they died. Participants were therefore less willing to take purebreds to mass vaccination campaigns than mongrels, but also reported that purebreds were more likely to be vaccinated by private veterinarians.

“Purebreds get infected at the campaigns, this is why many don't take them [to the campaigns].” (Woman, urban zone)

#### Mistrust in quality of vaccination services and vaccine

Concerns about quality of vaccination services and about the vaccines themselves were also raised, particularly in the urban area. People were concerned that the vaccines might have expired, that the cold chain had not been maintained, and that the vaccine itself was questionable. Participants were also worried that needles might be reused and that the dogs could get other diseases from dogs at the campaign.

Woman1: Once they told me they stuck the same needle in all the dogs.

Woman2: For all dogs. That makes us distrustful.

Woman3: I was scared after that too. (Urban zone)

Participants mentioned that people often **mistrust free or cheap services** because they think what is offered might be counterfeit or of low quality. Some participants also expressed concern that the vaccine could lose its efficacy due to sun exposure. In contrast with peri-urban participants, who did not allude to veterinarian services, in the urban areas, several mentioned preferring private veterinary clinics because they are more thorough (e.g. dogs also get checked for other health problems), keep their vaccines in refrigerators, clearly show that they use new needles, and have existing relationships with them and their dogs. Importantly, private vets offer the assurance that they have received extensive training to care for their dogs. Participants, particularly in the urban areas, also felt that mass campaign staff should wear identification cards or show documents that guarantee institutional support and their background (i.e. veterinarian, technician or fieldworker trained by MOH, etc.), to diminish mistrust in their technical capacity.

#### High perceived risk of rabies for dogs and families

Mainly in the urban area, but also in the peri-urban area, the main motivator for dog vaccination is fear of rabies. One participant mentioned that Mariano Melgar was in a state of emergency and thus canine vaccines are available most of the time in health centers. Some participants in the peri-urban area also mentioned that it was due to this “rumor of rabies” that more people went to vaccinate:

“People used to not care if their dog was vaccinated or not … but now there is a reason to vaccinate, here in Mariano Melgar which is the most infected district, so we must be very careful.” (Man, peri-urban area)

Many individuals, particularly in the peri-urban area, have experienced dog bites in the past three years. Only in the peri-urban area did participants mention that vaccination reduces their concerns of dealing with the responsibility to others if their unvaccinated dog might bite them.

“Yesterday they showed us a video of a person with rabies. I have also seen this and it is horrible, horrible. People would be more aware [if they saw this].” (Woman, urban area)

#### Logistical barriers

**Fig 2:**
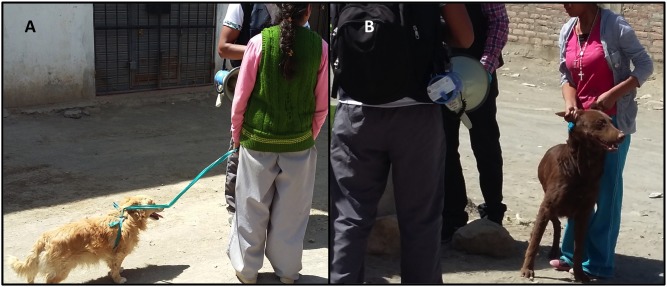
Community dwellers, not focus group participants, with their dogs at a rabies vaccination point in Mariano Melgar, February 2016. Note harness made of plastic bag (A) and lack of leash and holding dog by ears (B).

Logistical reasons were most discussed as the main barriers to dog vaccination. Participants from both areas mentioned **difficulty transporting dogs** to the vaccination sites as a primary reason for non-vaccination. This problem was exacerbated for those with more than one dog, large dogs, or dogs and/or owners with mobility issues. Particularly in the peri-urban areas, where most dogs have never used collars (compared to dogs in the urban areas), moving dogs is difficult: people came up with creative ways to pull their dogs, while others relied on brute force (Fig 2). Most participants in the peri-urban area and some in the urban area also described concerns about dogs escaping and fighting on the way to the vaccination campaign and at the vaccination point.

“If you pull a dog like this [dragging by ears or nape] and the dog is unknown in the area, all the other dogs come to it, they start fights with the unknown dogs. To avoid this and to try to escape, the dog pulls on you, and this is a problem when you are far away … if the dog is larger than you, you just have to let it go and find it [later]” (Man, urban area)

“My eldest son grabs his two front paws, and makes him walk…to the sport court. We rest when he can’t walk anymore; since he will not get into a car, we have to take him by force.” (Woman, peri-urban area)

### Interpersonal level factors

#### Social norms regarding relationship with dogs/pets and the animal care culture

When asked why people do not vaccinate their dogs, people from both areas cited a **lack of animal care culture** in Arequipa. Participants described that there are responsible, caring owners, but there are also indifferent, irresponsible or lazy owners who do not take their dogs to get vaccinated. It is important to note that no participants with unvaccinated dogs considered themselves to be a part of the latter group. Thus, all discussion about these “uncaring owners” was based on what participants thought of or perceived in other dog owners. Irresponsible or uncaring owners were described as viewing dogs as disposable objects. These owners keep dogs as guards; they do not have affectionate relationships with them and offer them only minimal care, feeding their dogs a couple of times per week in some cases.

“People are not responsible, they do not care what might happen with the doggies… They do not care if they die of hunger or cold. They only care about their own interests and protecting their houses. They do not have feelings for the doggy.” (Woman, peri-urban area)

Participants believe that if minimum care such as food and shelter is not provided by owners, then vaccination is unlikely. These types of dog owners were said to be mostly found in the higher parts of the peri-urban areas, where there is lack of enforcement of zoning codes, as evidenced by large stretches of land settled by pig farmers, and illegal land dealers coexisting with new settlers. Dogs in these areas roam freely, with minimum attention from their owners (e.g. they have to find much of their own food). These owners are unwilling to invest any time or money in the dogs, which are used only for protection.

“People here usually use dogs to protect the houses, but sometimes it gets out of control. They get a mutt female, she get pregnant on the street and then there are too many puppies to feed, so they start roaming the street and eating garbage.” (Man, peri-urban area)

Participants also discussed positive social norms regarding their relationships to their pets. Participants from both areas, but particularly from the urban area based on frequency and intensity of discussion, believe that people vaccinate their dogs because of their affection and sense of duty towards them: their pets are part of the family. They compared dogs with children in terms of their needs such as provision of affection, food, and care. Keeping dogs healthy is important also because it helps to guarantee the family’s safety, which was especially important for families with children. Only a few participants—all under 25 years old—in both areas made direct reference to animal rights and discussed animal care from an ethical perspective:

“I vaccinate them because I feel affection for them, I do not pay much attention to the rabies aspect. Everyone thinks about the people, but the animal´s life also counts.” (Woman, peri-urban area)

#### Social pressure from the community to vaccinate or not

When discussing whether there was social pressure in the communities to vaccinate, participants described those who didn’t vaccinate as individualistic and unconcerned about the effect on the community. Some participants described questioning neighbors about their unvaccinated dogs and said that they received evasive and even aggressive responses.

Interviewer: Have you ever asked [neighbors who don’t vaccinate their dogs], ´Why don’t you vaccinate your dog if there is rabies?

Woman1: They say ´I don’t have the time, but if you want, you take [the dog] for me…

Woman2: ´[Not vaccinating my dog] is not your problem´ they say. (Peri-urban area)

Some participants even mentioned that their neighbors and peers make fun of them for vaccinating their dogs:

“…they argue with you, they say ´you are a fool, you are wasting money.” (Woman, urban zone)

*“*Participants perceive that their neighbors’ decision not to vaccinate their dogs is influenced by other more urgent needs that they cannot meet because of lack of means or time, for example, their children’s medical care or their need to work:

“´If I don’t even vaccinate my kids, you think I am going to vaccinate my dog?´ they say.” (Woman, peri-urban area)

One participant mentioned that people who do not own animals should also be part of the discussion and raise their voices within their communities to promote rabies vaccination, rather than waiting until a dog bites to take action. Others mentioned that pressure to vaccinate sometimes comes from other people, such as family or veterinarians, and that people can promote vaccination within their circle of friends and neighbors:

“I was motivated by my mother, she told me ´Take him. It could happen to him and you haven't vaccinated him, it could be dangerous´.” (Man, urban area)

### Organizational factors

#### Insufficient health promotion and communication for dog vaccination campaigns

Most participants who knew about the campaign learned about it through radio and megaphone announcements. Participants in both areas reported **difficulty understanding the megaphone announcements**, and that they only reached people at home at the time of the announcements, excluding those who work all day. In the peri-urban area they also explained that the cars do not drive up to the higher areas where there are no roads. Radio was perceived to be a preferred channel because it has a broader reach. A few people said that visual information such as posters and flyers prior to the campaign could improve awareness. Many in the urban area also mentioned hearing about the campaign through television ads, whereas some in the peri-urban areas heard about it through door-to-door health promoters, messages to parents and children at school meetings, and community boards and assemblies. The **timing of the messages** is also important: some found out about the campaign at the last minute through word of mouth or by seeing people taking dogs to the campaign. This untimely information did not allow them to prepare to participate, particularly if the date and location of the campaign was inconvenient for them.

Participants from both areas also discussed the **types of messages** that should be diffused. Some suggested using “threatening” messages to get people to vaccinate, for example, with the threat of sanctions or fines, and door-to-door mandatory vaccination visits. In the urban area, some people suggested focusing the awareness campaigns on the family’s or children’s health:

“I think that the focus should be on the family, because if you aim it at the dog, no. [They should say] ´Avoid your child's suffering´.” (Woman, urban zone)

Some participants in both areas spontaneously remembered the Chagas campaign [48] (a door-to-door indoor residual spraying strategy) as an effective program. They described the Chagas campaign as meticulous in implementation (health workers asked and recorded details about the animals, the house, etc.), insistent (they left notifications and would return if the task was not completed), mandatory (there was no way of denying the spray, people would get fined) and informative (there was publicity, and people learned about the illness and prevention measures). All these factors created trust in the quality of the program.

“The only campaign I’ve seen that has come to my house up to two or three times is Chagas. I´ve seen they care a lot about coming once, twice, if you´re not home, they come back.” (Woman, urban zone)

#### Inadequate location and low frequency of mass vaccination campaign

The regular mass vaccination campaigns occur once a year on a Sunday or a weekend; however, for study participants even twice a year is too sparse. People felt the vaccination campaigns should be **carried out more frequently**, so that if you had to miss one campaign, you could still make another; participants also recommend having campaigns on different days, since market activity (many people in the peri-urban area work in the markets) is highest on weekends.

Several people mentioned that **door-to-door campaigns** would facilitate the vaccination process for many, but otherwise, community areas that have high pedestrian traffic, with ample space, kiosks, and other facilities (i.e. bathrooms) should be prioritized, such as sports courts, boardwalks, or parks. A few participants in the urban area mentioned that the health posts and main squares were good places for dog vaccination because they are typically well-known and there is usually one in each community. In the peri-urban area, people suggested other locations, such as the numerous game courts (multi-sport athletic space, typically constructed outdoors with concrete floor), which are known by the community and offer ample space to handle the dogs brought to the vaccination point.

“For example, Atalaya only does [the campaign] down below, … people up here won´t go down there. If they did it in all the game courts I know people would go and there would be fewer unvaccinated dogs.” (Man, peri-urban)

Even though a door-to-door campaign was suggested by some, a few peri-urban participants said that this strategy would not increase participation among individuals who keep dogs primarily to provide safety for their properties. Such non-participating individuals, many of whom are squatters on their land, are not often at home.

#### Limited personnel vaccinating during the campaign

Participants believe the limited number of staff and the consequent **long lines** at vaccination points contribute to the risk of **dogs fighting**.

“When I was separating my dog from the others that were attacking him [at the vaccination site], another dog came and, boom, bit my hand” (Woman, peri-urban area)

To avoid the lines and dogfights, some people have tried to go very early or at the end of the day, even though they risk missing the vaccination campaign.

Participants felt more vaccination personnel should be hired to reduce disorganization. In both areas, participants also thought that additional personnel at these campaigns could provide an opportunity to offer other services to dogs, such as deworming and checkups.

### Community factors

#### Distance to vaccination point area and difficult topography

Distance to vaccination points was reported as a barrier. Taking dogs for **more than a few blocks was inconvenient** for some participants; they felt more people would go if there were vaccination points at more locations.

Interviewer: What the maximum distance you would walk [to the vaccination point]?

Woman: Let’s say 4, 5 blocks.

Interviewers: And you?… how long would you be willing to walk?

Man: 5, 6 blocks is ok. (Peri-urban area)

"Walking 8 blocks with 3 doggies, come on, that’s a lot" (Woman, peri-urban area).

Topography and the city layout also play a role in access to the vaccination points. In the urban area some people considered crossing large avenues with their dogs to be dangerous, while those from the peri-urban area described difficulty walking up or down from the higher parts because of the unlevel ground.

“I walked 5 blocks [to vaccinate my dog], but it was very difficult, because it is uphill and very steep.” (Woman, peri-urban area)

Interviewer: How many blocks would you walk your dogs?

Woman: 2 or 3 blocks maximum … crossing an avenue the car almost hit my dog. I am telling you that I suffered to take him [to the vaccination point] because the taxis didn’t want to bring me. Could you imagine if I have to walk up the hill [to the vaccination point]? (Urban area)

#### Local security and poor housing materials

In both urban and peri-urban areas we found some participants own dogs for protection; however, the perception of security was different between the two areas. Participants from the peri-urban area mentioned thieves getting easy access to their houses. They explained that in their communities several dogs are needed to provide home security, but the presence of many guard dogs in the area impedes pedestrian traffic and prevents people from taking dogs to the vaccination campaign.

Woman: I have to walk like a crazy woman, with a stone in my hand, because there are five dogs at one house, more dogs in another house…

Man: And the dogs up here in Atalaya, are not small, the majority are huge dogs. (Peri-urban area)

Compared to urban houses, in the peri-urban area houses are built with poorer materials: fences are created with stacked bricks or stones and doors and roofs are built with corrugated metal, which cannot keep dogs in or out; dogs can knock down walls or tear corrugated doors open to access the street. Equally problematic, stray dogs can enter houses, and try to do so when there is a female in heat. In the peri-urban areas, dog aggressiveness, density (more dogs per household), and lack of restraints (collars or leashes) result in increased difficulty to get them to the campaign: dogs may not be at home during the campaign, and dog owners may not be able to handle these more numerous and aggressive animals.

“They don’t want female dogs, because… when they are in heat and within the house, the stray dogs get in the house even from the roof” (Woman, peri-urban area)

Also in the peri-urban area, dogs are less likely to use a collar or have walked on a leash with their owners. The task of putting a collar and leash, if there is one available, and walking a dog once a year to the vaccination point is a strange and even unsettling event for both dog and owner.

“I haven’t vaccinated my dogs yet. I don't keep them on a leash either, but if I were to take them to vaccinate, I would have to pull them with a rope… I don't know” (Woman, peri-urban area).

## Discussion

Various theories have been developed to guide assessments of effectiveness of rabies vaccination campaigns [10,52,53]; an important component of this work is examining the perspectives of dog owners and community members, both qualitatively and quantitatively [53]. This article analyzes unique focus group data, and is the first from our ongoing multi-method studies of factors associated with coverage of and participation in mass canine rabies vaccination campaigns. The social ecological model [9] applied here to structure the themes raised by FG participants is a theory-based model that recognizes the complexity of the socio-cultural system in which individuals make decisions and take actions. Our findings reveal multiple barriers to dog vaccination at the **individual, interpersonal, organizational** and **community levels** of the social ecological model.

We identified **individual**-level barriers to vaccination as well as facilitators of vaccination. An important next step is to translate these barriers and facilitators into proposed solutions to improve campaign effectiveness, using evidence-based concepts from health behavior change theory. For example, our respondents suggested using fear-based promotional materials and punishments for non-participation. This proposed solution highlights the importance of self-efficacy (the self-perceived ability that one can practice a proposed solution) as a driver of health behavior, as posited in several health behavior change theories, including the extended parallel processing model (EPPM) [54]. The EPPM model proposes that when faced with a health risk, people will take actions to protect their health if they are empowered by an appropriate balance of fear and self-efficacy [54], consistent with other models that predict adverse consequences from employing fear in health communication programs without assuring high self-efficacy levels [55–58]. A health communication campaign that focuses on instilling fear alone will often result in a feeling of helplessness and inaction. However, communication that conveys risk in an understandable way and also provides a clear and do-able call to action to reduce one’s risk leads to empowered citizens who take action [59].

Rabies is a fatal and devastating disease and Arequipa is facing a sustained rabies outbreak that has not yet been controlled, posing a serious risk for humans and animals; vaccinating one’s dog is a highly effective prevention method that only requires dog owners’ efforts once a year. Rabies vaccination communication should tap into this feeling of risk (to increase individuals’ perceived susceptibility to rabies, and inform them of the severity of this virus), while at the same time help individuals feel confident in being able to act (self-efficacy in handling their dogs and participating in campaigns) to effectively mitigate that risk. This communication should not only address knowledge gaps, but also inspire trust in the program and emphasize motivators that participants described, including keeping one’s family healthy, protecting the household pets from rabies, and keeping the community free of rabies.

A very salient individual level barrier mentioned in FG was the logistics: difficulty getting dogs to vaccination sites, dog fights en route to and at the vaccination points. To a certain extent, this barrier can also be mitigated through improvements to the rabies vaccination program. Other Latin American countries have taken steps to improve people’s ability to handle their dogs. For instance, Costa Rica included in their “Guidelines of Pet Reproduction and Ownership” to walk dogs on the leash frequently to increase their proper socialization [60], and new Mexican bills support citizens’ initiatives to adequately integrate dogs in society, such as the “Social Dog, Responsible Owner Program” [61], which provides training to dog owners and their dogs through in-person workshops, online videos, and written material. Based on our findings, to address some of the knowledge gaps in the population that could deter vaccination for some dogs, health communication campaigns should include additional information about when one should vaccinate one’s dogs and describing that all dogs (even those that are always indoors) are at risk.

At the **interpersonal level**, barriers discussed by participants were related to the social norms of poor animal care culture in Arequipa, competing social pressure both for and against vaccination, although the latter was considerably less common. Most respondents in this study also indicated that there is social pressure in Arequipa for people to vaccinate dogs, as those who do not vaccinate are seen in a negative light; they are considered lazy, uncaring for animals, and irresponsible neighbors in the context of community protection offered by vaccines (although a few also described social pressure to not vaccinate—feeling laughed at for spending time and/or money on a dog). The theories of diffusion of innovations [62], social learning [63] and planned behavior and reasoned action [64] point to the importance of social norms, the opinion of others, and social networks in making decisions about health behavior. Vaccine promotion communication in Arequipa should be informed by these theories and seek to influence local norms by providing information from trusted and influential sources, as well as identifying well-connected individuals in communities to champion certain ideas—including but not limited to local storekeepers, health workers that routinely work in certain areas, health care providers (i.e. veterinarians), local neighborhood authorities, municipal governments, health officials, local celebrities, teachers, and respected elders.

Barriers that emerged at the **organizational level** were health system weaknesses, such as insufficient promotion of the vaccination campaign, poor selection of location for the vaccination points, low frequency of vaccination campaigns, and insufficient staff during the campaign. Regarding the service, trust in quality of services can be improved through strategic decisions and actions in the implementation phase. Identification cards with the vaccinator’s name and qualifications should be visible to campaign attendees—both on the vaccinator and on any visible media (e.g. posters, banners) at the site. Likewise, keeping coolers visible and less exposed to the sun might help increase confidence that the cold chain has been kept intact, showing the unopened syringe or needles before filling them would quell rumors of syringes are reused, and pointing out the expiration date on the vaccine vials before filling the syringe would take seconds, but increase trust in the product. Finally, a barrier mentioned in many FG was the *insufficient or untimely information about date and locations* of vaccine campaigns. This barrier can also be addressed in the planning and implementing phases: announcements about the campaign can start earlier and through different media, and be conducted on different weekdays and times, to ensure more people hear about them. Posting times, dates, and locations of upcoming vaccination campaigns in advance and in visible community locations would also allow for individuals to plan where and when to take their dogs for the vaccination, and to plan to have extra people to help take the dogs if necessary. Participants mentioned other health programs based on door-to-door interventions such as the Chagas control campaign that could have lessons to offer the rabies campaign. Door-to-door strategies have been discussed among local officials to promote participation and also enable a simultaneous canine census to improve the accuracy of coverage data. These and other interventions at the organizational level to improve rabies vaccination uptake can be modeled according to the health care access framework presented by Obrist et al. [52]. This framework consists of five dimensions: availability, accessibility, affordability, adequacy, and acceptability; the present study provides insights into each of these dimensions.

At the **community level,** barriers cited included distance to vaccination points, difficult topography (particularly in the peri-urban areas), and lack of security in the area. Flexible strategies are necessary to meet the local community needs [11,16]; for instance, in rural Tanzania [11], a few central-point canine rabies vaccination points were insufficient to achieve 70% coverage (WHO recommended threshold), and had to be supplemented by door-to-door efforts [65]. The combination of central-point and door-to-door dog vaccination has also proven to be effective in urban environments, with one large city recently achieving 79% coverage [66]. Participants’ preference for door-to-door campaigns is also important to highlight: this has usually been done in rural areas [11,66], and although the topography and urban design in some cities is ideal for centralized vaccinations, clearly not all urbanized settings are the same: a central-point mass vaccination campaign in the urban areas of N’Djamena, Chad achieved a coverage above 70% [67], while a similar central-point vaccination campaign in the city of Bamako, Mali achieved only 17% coverage [10]. In our study, campaigns in the urban areas were fairly accessible to dog owners (despite having to cross large avenues), but the topography of the peri-urban areas was much more difficult (steep hills, large rocks, unpaved roads) for people trying to get multiple unleashed dogs to vaccination points. Rapid urban spread, low salaries, scarce resources and bureaucratic rules can all impede the responsiveness and quality of campaign services [68]. For rabies, control programs must take into account social, political and cultural contexts to improve efficacy and avoid the barriers faced by other top-down public health interventions [69–71].

Our study and others [16–18,26,72–74] have focused on dog owners’ knowledge, dog-ownership practices, norms, and perceptions about the dog vaccination campaign as important factors to understand the rabies program’s outcomes. But capacities, norms, and policies of implementing institutions also play a central role in rabies control efforts [16]. Overall, although some of the suggestions discussed would add to program expenses, several imply little marginal cost; many suggested strategies simply optimize current investments for better efficiency. For example, current promotional materials could be more informative (i.e. include location and hours of vaccinations) and be disseminated earlier; vaccinators could visibly display their ID and credentials; and vaccine syringes could be opened in front of dos owners with additional explanations about safety and quality procedures to increase trust in the campaign.

Similar to prior studies [74], we found different attitudes, dog-ownership practices, and knowledge levels between urban and peri-urban areas. Given the different characteristics of these areas (topography, migration history, level of urbanization, levels of security and utilization of dogs for companionship vs. protection), it is difficult to justify a one-size-fits-all campaign strategy. The newer peripheral communities we studied in Arequipa are typical of peri-urban areas, a neglected zone in Latin America, Africa, and Asia [75] commonly characterized by inadequate infrastructure, service provision, and land tenure security [38,76]. While most public health campaigns have distinct strategies for urban vs. rural areas; the peri-urban area (or rural-urban interface), one of the fastest growing areas in the world [75], lacks a specific and responsive programmatic approach in Peru and elsewhere.

## Conclusions

Important individual, interpersonal, organizational, and community level factors limit more widespread participation in dog rabies vaccination campaigns in the city of Arequipa–site of a canine rabies outbreak since 2015. A comprehensive communication campaign is required to increase the population’s knowledge about rabies’ transmission, its consequences, and prevention measures. Developing strategies to help dog owners not accustomed to using leashes should be explored and implemented to help facilitate the transport of dogs to vaccination points. It is important to provide timely information about free dog mass vaccination campaigns. Clear identification of vaccinators and demonstration of unopened needles and syringes, as well as training vaccinators on their interactions with dog owners and response to their concerns can also help increase trust in the campaign. Finally, flexible strategies are needed to serve diverse communities within a city; urban and peri-urban areas present contrasting landscapes that might require different vaccine point locations.

## Supporting information

S1 Abstract Resumen en español (Alternative language abstract). (PDF)

## References

[1] Pan American Health Organization, World Health Organization. Epidemiological Alert [Internet]. 2015 Jun. Available: http://www.paho.org/hq/index.php?option=com_docman&task=doc_download&Itemid=&gid=30659&lang=en

[2] Ministerio de Salud del Peru—Direccion General de Epidemiologia. Alerta Epidemiológica [Epidemiologic Alert]. Lima; 2015. Report No.: AE-DEVE N. 003–2015.

[3] El Presidente de la República. Decreto Supremo que declara en Emergencia Sanitaria por el plazo de noventa (90) días calendario, a la provincia de Arequipa del departamento de Arequipa [Internet]. Apr 2, 2016 pp. 582224–582229. Available: http://busquedas.elperuano.com.pe/normaslegales/decreto-supremo-que-declara-en-emergencia-sanitaria-por-el-p-decreto-supremo-n-015-2016-sa-1363166-5/

[4] Knobel DL, Lembo T, Morters M, Townsend SE, Cleaveland S, Hampson K. Dog Rabies and Its Control. Rabies. Elsevier; 2013 pp. 591–615.

[5] Vigilato MAN, Clavijo A, Knobl T, Silva HMT, Cosivi O, Schneider MC, et al Progress towards eliminating canine rabies: policies and perspectives from Latin America and the Caribbean. Philos Trans R Soc Lond, B, Biol Sci. 2013;368: 20120143 10.1098/rstb.2012.0143 23798691 PMC3720041

[6] Hampson K, Coudeville L, Lembo T, Sambo M, Kieffer A, Attlan M, et al Estimating the global burden of endemic canine rabies. PLoS Negl Trop Dis. 2015;9: e0003709 10.1371/journal.pntd.0003709 25881058 PMC4400070

[7] Lembo T, Hampson K, Kaare MT, Ernest E, Knobel D, Kazwala RR, et al The feasibility of canine rabies elimination in Africa: dispelling doubts with data. Rupprecht CE, editor. PLoS Negl Trop Dis. 2010;4: e626 10.1371/journal.pntd.0000626 20186330 PMC2826407

[8] Tenzin Ward MP. Review of Rabies Epidemiology and Control in South, South East and East Asia: Past, Present and Prospects for Elimination. Zoonoses Public Health. 2012;59: 451–467. 23180493 10.1111/j.1863-2378.2012.01489.x

[9] Bronfenbrenner U. Ecological models of human development. International Encyclopedia of Education. 2nd ed. 1994.

[10] Muthiani Y, Traoré A, Mauti S, Zinsstag J, Hattendorf J. Low coverage of central point vaccination against dog rabies in Bamako, Mali. Prev Vet Med. 2015;120: 203–209. 10.1016/j.prevetmed.2015.04.007 25953653

[11] Kaare M, Lembo T, Hampson K, Ernest E, Estes A, Mentzel C, et al Rabies control in rural Africa: Evaluating strategies for effective domestic dog vaccination. Vaccine. 2009;27: 152–160. 10.1016/j.vaccine.2008.09.054 18848595 PMC3272409

[12] Lapiz SMD, Miranda MEG, Garcia RG, Daguro LI, Paman MD, Madrinan FP, et al Implementation of an intersectoral program to eliminate human and canine rabies: the Bohol Rabies Prevention and Elimination Project. Zinsstag J, editor. PLoS Negl Trop Dis. 2012;6: e1891 10.1371/journal.pntd.0001891 23236525 PMC3516573

[13] Kayali U, Mindekem R, Yemadji N. Coverage of pilot parenteral vaccination campaign against canine rabies in N'Djamena, Chad. Bull World Health Organ 2003; 81(10): 739–44. 14758434 PMC2572337

[14] Durr S, Mindekem R, Kaninga Y, Doumagoum Moto D, Meltzer MI, Vounatsou P, et al Effectiveness of dog rabies vaccination programmes: comparison of owner-charged and free vaccination campaigns. Epidemiol Infect. 2009;137: 1558–1567. 10.1017/S0950268809002386 19327197

[15] Minyoo AB, Steinmetz M, Czupryna A. Incentives Increase Participation in Mass Dog Rabies Vaccination Clinics and Methods of Coverage Estimation Are Assessed to Be Accurate. PLoS Negl Trop Dis. 2015;9(12): e0004221 10.1371/journal.pntd.0004221 26633821 PMC4669116

[16] Bardosh K, Sambo M, Sikana L, Hampson K, Welburn SC. Eliminating rabies in Tanzania? Local understandings and responses to mass dog vaccination in Kilombero and Ulanga districts. Rupprecht CE, editor. PLoS Negl Trop Dis. 2014;8: e2935 10.1371/journal.pntd.0002935 24945697 PMC4063706

[17] Serebe SG, Tadesse KA. Study on community knowledge, attitude and practice of rabies in and nearby Gondar town, North West Ethiopia. Journal of Public and Epidemiology. 2014; 6(12): 429–35.

[18] Kabeta T, Deresa B, Tigre W, Ward MP, Mor SM. Knowledge, Attitudes and Practices of Animal Bite Victims Attending an Anti-rabies Health Center in Jimma Town, Ethiopia. Zinsstag J, editor. PLoS Negl Trop Dis. 2015;9: e0003867 10.1371/journal.pntd.0003867 26114573 PMC4482645

[19] De Ramos M, Bravo LC. Knowledge, attitudes, and practices of community regarding animals bite and rabies. Paediatric Infectious Disease Society of the Philippines Journal. 2004; 8(1): 24–32 http://www.pidsphil.org/pdf/Journal_05210731/jo25_ja04.pdf

[20] Singh US, Choudhary SK. Knowledge, attitude, behavior and practice study on dog-bites and its management in the context of prevention of rabies in a rural community of Gujarat. Indian J of Community Med. 2005;81–3.

[21] Bandura A. Health promotion by social cognitive means. Health education & behavior. 2004; 31(2): 143–64. 15090118 10.1177/1090198104263660

[22] Benyoussef A, Cutler JL, Levine A, Mansourian P, Phan-Tan T, Baylet R, et al Health effects of rural-urban migration in developing countries—Senegal. Soc Sci Med. 1974;8: 243–262. 4852227 10.1016/0037-7856(74)90093-6

[23] Li Y. Understanding health constraints among rural-to-urban migrants in China. Qual Health Res. 2013;23: 1459–1469. 10.1177/1049732313507500 24122513

[24] Rosenstock IM. Why people use health services. Milbank Q. 2005; 84(4).

[25] Zhang L, Li X, Yang H, Mao R, Zhao Q. Health status and health-seeking behaviour between interprovincial and intraprovincial rural-to-urban young migrants in Nanjing China. Asia-Pacific Population Journal. 2011;26: 39–54.

[26] Widyastuti MDW, Bardosh KL, Sunandar, Basri C, Basuno E, Jatikusumah A, et al On dogs, people, and a rabies epidemic: results from a sociocultural study in Bali, Indonesia. Infectious Diseases of Poverty. 2015;4: 60. 26137295 10.1186/s40249-015-0061-1 PMC4486702

[27] Ensor T. Overcoming barriers to health service access: influencing the demand side. Health Policy and Planning. 2004;19: 69–79. 14982885 10.1093/heapol/czh009

[28] Kiwanuka SN, Ekirapa EK, Peterson S, Okui O, Rahman MH, Peters D, et al Access to and utilisation of health services for the poor in Uganda: a systematic review of available evidence. Trans R Soc Trop Med Hyg. 2008;102: 1067–1074. 10.1016/j.trstmh.2008.04.023 18565559

[29] Jacobs B, Ir P, Bigdeli M, Annear PL, Van Damme W. Addressing access barriers to health services: an analytical framework for selecting appropriate interventions in low-income Asian countries. Health Policy and Planning. 2012;27: 288–300. 10.1093/heapol/czr038 21565939

[30] Tweheyo R, Konde-Lule J, Tumwesigye NM, Sekandi JN. Male partner attendance of skilled antenatal care in peri-urban Gulu district, Northern Uganda. BMC Pregnancy Childbirth. 2010;10: 53 10.1186/1471-2393-10-53 20846369 PMC2946269

[31] Brook RK, Kutz SJ, Millins C, Veitch AM. Evaluation and delivery of domestic animal health services in remote communities in the Northwest Territories: a case study of status and needs. Can Vet Journal 2010. PMC2942049 21197203

[32] Ogun AA, Okonko IO, Udeze AO. Feasibility and factors affecting global elimination and possible eradication of rabies in the world. J Gen Mol Virol. 2010; 2(1): 1–27

[33] Dirección General de Salud de las Personas, Dirección de Atención Integral de Salud Norma Técnica de Salud para la Prevención y Control de la Rabia Humana en el Perú [Technical Health Norm for the Prevention and Control of Human Rabies in Peru] [Internet]. 1st ed. Lima, Peru: Ministerio de Salud; 2006 p. 101 Available: http://www.minsa.gob.pe/portalweb/06prevencion/est_san/archivo/2011/NTS_DE_RABIA.pdf

[34] Downes MJ, Dean RS, Stavisky JH, Adams VJ, Grindlay DJC, Brennan ML. Methods used to estimate the size of the owned cat and dog population: a systematic review. BMC Vet Res. 2013;9: 121 10.1186/1746-6148-9-121 23777563 PMC3689088

[35] Grace D, Mutua F, Ochungo P, Kruska R, Jones K, Brierley L, et al. Mapping of Poverty and Likely Zoonoses Hotspots. Zoonoses Project 4. Report to the UK Department for International Development. Nairobi, Kenya: ILRI; 2012.

[36] Institute of Medicine US Forum on Microbial Threats. Neglected Zoonotic Diseases In: King L, editor. The Causes and Impacts of Neglected Tropical and Zoonotic Diseases: Opportunities for Integrated Intervention Strategies. Washington (DC): National Academies Press (US); 2011. 21977543

[37] LeJeune J, Kersting A. Zoonoses: an occupational hazard for livestock workers and a public health concern for rural communities. J Agric Saf Health. 2010;16: 161–179. 20836437 10.13031/2013.32041

[38] Levy MZ, Barbu CM, Castillo-Neyra R, Quispe Machaca VR, Ancca Juárez J, Escalante-Mejia P, et al Urbanization, land tenure security and vector-borne Chagas disease. Proc Biol Sci. 2014;281: 20141003 10.1098/rspb.2014.1003 24990681 PMC4100517

[39] Day MJ, Breitschwerdt E, Cleaveland S, Umesh K, Khanna C, Kirpensteijn J, et al Surveillance of Zoonotic Infectious Disease Transmitted by Small Companion Animals. Emerging Infect Dis. Centers for Disease Control and Prevention; 2012;18: e1.

[40] Amitai Z, Bromberg M, Bernstein M. A large Q fever outbreak in an urban school in central Israel. Clin Infect Dis. 2010;50: 1433–1438. 10.1086/652442 20415568

[41] Liu Q, Cao L, Zhu X-Q. Major emerging and re-emerging zoonoses in China: a matter of global health and socioeconomic development for 1.3 billion. Int J Infect Dis. 2014;25: 65–72. 10.1016/j.ijid.2014.04.003 24858904 PMC7110807

[42] Neiderud C-J. How urbanization affects the epidemiology of emerging infectious diseases. Infect Ecol Epidemiol. 2015;5: 27060 10.3402/iee.v5.27060 26112265 PMC4481042

[43] Reyes MM, Taramona CP, Saire-Mendoza M, Gavidia CM, Barron E, Boufana B, et al Human and canine echinococcosis infection in informal, unlicensed abattoirs in Lima, Peru. PLoS Negl Trop Dis. 2012;6: e1462 10.1371/journal.pntd.0001462 22509413 PMC3317905

[44] Singh BB, Sharma R, Gill JPS, Aulakh RS, Banga HS. Climate change, zoonoses and India. Rev—Off Int Epizoot. 2011;30: 779–788. 22435190 10.20506/rst.30.3.2073

[45] Wallace RM, Reses H, Franka R, Dilius P, Fenelon N, Orciari L, et al Establishment of a Canine Rabies Burden in Haiti through the Implementation of a Novel Surveillance Program. PLoS Negl Trop Dis. 2015;9: e0004245 10.1371/journal.pntd.0004245 26600437 PMC4657989

[46] Widdowson M-A, Morales GJ, Chaves S, McGrane J. Epidemiology of urban canine rabies, Santa Cruz, Bolivia, 1972–1997. Emerging Infect Dis. 2002;8: 458–461. 10.3201/eid0805.010302 11996678 PMC2732486

[47] Instituto de Estadística e Informática Perú. Población y Vivienda. Estimaciones y Proyecciones de Población. Población total al 30 de junio del 2013, por grupos quinquenales de edad, según departamento, provincia y distrito. [Internet]. Lima, Perú: INEI; 2008 Available: https://www.inei.gob.pe/estadisticas/indice-tematico/poblacion-y-vivienda/

[48] Buttenheim AM, Paz-Soldan V, Barbu C, Skovira C, Quintanilla Calderón J, Mollesaca Riveros LM, et al Is participation contagious? Evidence from a household vector control campaign in urban Peru. J Epidemiol Community Health. 2014;68: 103–109. 10.1136/jech-2013-202661 24062411 PMC3888816

[49] Bernard HR. Research Methods in Anthropology. 4 ed. Rowman Altamira; 2006.

[50] Muhr T. ATLAS.ti 6.0. 6 ed. Berlin, Germany: Scientific Software Development GmbH; 2008.

[51] Injury Prevention and Control: Division of Violence Prevention The social-ecological model: A framework for prevention. In: Centers for Disease Control and Prevention [Internet]. Atlanta, USA: National Center for Injury Prevention …; [cited 25 Sep 2016]. Available: http://www.cdc.gov/violenceprevention/overview/social-ecologicalmodel.html

[52] Obrist B, Iteba N, Lengeler C, Makemba A, Mshana C, Nathan R, et al Access to Health Care in Contexts of Livelihood Insecurity: A Framework for Analysis and Action. PLoS Med. 2007;4: e308. 10.1371/journal.pmed.0040308 PMC2039761 17958467

[53] Mosimann L, Traoré A, Mauti S, Léchenne M, Obrist B, Véron R, et al A mixed methods approach to assess animal vaccination programmes: The case of rabies control in Bamako, Mali. Acta Tropica. 2017;165: 203–215. 10.1016/j.actatropica.2016.10.007 27751865

[54] Witte K. Putting the fear back into fear appeals: The extended parallel process model. Communication Monographs. 1992;59: 329–349.

[55] Soames Job RF. Effective and ineffective use of fear in health promotion campaigns. Am J Public Health. 1988;78: 163–167. 3276236 10.2105/ajph.78.2.163 PMC1349109

[56] Cho H, Salmon CT. Unintended Effects of Health Communication Campaigns. J Commun. 2007;57: 293–317.

[57] Witte K. The role of threat and efficacy in AIDS prevention. Int Q Community Health Educ. 1992;12: 225–249. 10.2190/U43P-9QLX-HJ5P-U2J5 20840971

[58] Janis IL, Feshbach S. Effects of fear-arousing communications. The Journal of Abnormal and Social Psychology. 1953;48: 78–92. 10.1037/h0060732 13022199

[59] Rimal RN, Real K. Perceived Risk and Efficacy Beliefs as Motivators of Change: Use of the Risk Perception Attitude (RPA) Framework to Understand Health Behaviors. Human Communication Research. 2003;29: 370–399.

[60] El Presidente de la República, Ministerio de Salud de Costa Rica. Regulation of Pet Reproduction and Ownership [Reglamento para la Reproducción y Tenencia Responsable de Animales de Compañía] [Internet]. Sep 22, 2003. Available: http://www.poder-judicial.go.cr/violenciaintrafamiliar/index.php/normativa/category/36-reglamentos%3Fdownload%3D345:reglamento-reproduccion-tenencia-responsable-animales

[61] Rodriguez Medonza J de J. Bill to Amend the Law on Protection and Dignified Treatment of Animals for the State of Coahuila [Proyecto de Modificacion de la Ley de Protección y Trato Digno a los Animales para el Estado de Coahuila] [Internet]. Comisión de Salud, Medio Ambiente, Recursos Naturales y Agua, editor. Available: http://congresocoahuila.gob.mx/portal/wp-content/uploads/2014/11/20160420_263_PVEM.doc

[62] Rogers EM. Diffusion of Innovations. 4 ed. New York: The Free Press; 1995.

[63] Bandura A. Social learning theory. Englewood Cliffs, NJ: Prentice-Hall; 1977.

[64] Ajzen I. The theory of planned behavior. Organizational Behavior and Human Decision Processes. 1991;50: 179–211.

[65] World Health Organisation. WHO Expert Consultation on Rabies. Second report. World Health Organ Tech Rep Ser. 2013;: 1–139– back cover. 24069724

[66] Gibson AD, Handel IG, Shervell K, Roux T. The Vaccination of 35,000 Dogs in 20 Working Days Using Combined Static Point and Door-to-Door Methods in Blantyre, Malawi. PLoS Negl Trop Dis. 2016; 10(7): e0004824 10.1371/journal.pntd.0004824 27414810 PMC4945057

[67] Léchenne M, Oussiguere A, Naissengar K, Mindekem R, Mosimann L, Rives G, et al Operational performance and analysis of two rabies vaccination campaigns in N’Djamena, Chad. Vaccine. 2016;34: 571–577. 10.1016/j.vaccine.2015.11.033 26631415

[68] Rifkin SB. Paradigms lost: toward a new understanding of community participation in health programmes. Acta Tropica. 1996;61: 79–92. 8740887 10.1016/0001-706x(95)00105-n

[69] Hahn RA, Inhorn M. Anthropology and Public Health. London: Oxford University Press; 2008.

[70] Matland RE. Synthesizing the implementation literature: The ambiguity-conflict model of policy implementation. Journal of public administration research and theory. 1995.

[71] Potvin L, Gendron S, Bilodeau A, Chabot P. Integrating social theory into public health practice. Am J Public Health. 2005;95: 591–595. 10.2105/AJPH.2004.048017 15798114 PMC1449225

[72] Dürr S, Meltzer MI, Mindekem R, Zinsstag J. Owner valuation of rabies vaccination of dogs, Chad. Emerging Infect Dis. 2008;14: 1650–1652. 10.3201/eid1410.071490 18826838 PMC2609875

[73] Sambo M, Lembo T, Cleaveland S, Ferguson HM, Sikana L, Simon C, et al Knowledge, Attitudes and Practices (KAP) about Rabies Prevention and Control: A Community Survey in Tanzania. Rupprecht CE, editor. PLoS Negl Trop Dis. 2014;8: e3310 10.1371/journal.pntd.0003310 25473834 PMC4256472

[74] Tenzin, Dhand NK, Rai BD, Changlo, Tenzin S, Tsheten K, et al Community-based study on knowledge, attitudes and perception of rabies in Gelephu, south-central Bhutan. International Health. 2012;4: 210–219. 10.1016/j.inhe.2012.03.005 24029402

[75] Douglas I. Peri-Urban Ecosystems and Societies: Transitional Zones and Contrasting Values. In: McGregor D, Simon D, Thompson D, editors. The Peri-Urban Interface. London; 2006. p. 336.

[76] Ooi GL, Phua KH. Urbanization and slum formation. J Urban Health. 2007;84: i27–34. 10.1007/s11524-007-9167-5 17387618 PMC1891640

