# Supplementary material for: Barriers to dog rabies vaccination during an urban rabies outbreak: Qualitative findings from Arequipa, Peru
Source: PLoS Negl Trop Dis. 2017 Mar 17;11(3):e0005460. doi: 10.1371/journal.pntd.0005460 (PMC5371379; doi:10.1371/journal.pntd.0005460)
Supplement: S1 Abstract — (PDF) [file pntd.0005460.s001.pdf]

Supporting material 1. Spanish Abstract

Resumen

Antecedentes
La rabia canina se ha reintroducido en la ciudad de Arequipa, Perú en marzo del 2015. El Ministerio de Salud ha realizado una seria de campañas masivas de vacunación canina para contener el brote, pero la transmisión del virus de rabia canina continua en el complejo ambiente urbano de Arequipa, poniendo en riesgo al casi millón de habitantes de la ciudad. El factor proximal de la rabia canina en Arequipa es la baja tasa de vacunación canina. Nuestros objetivos fueron estimar cualitativamente las barreras y facilitadores de la vacunación de perros contra la rabia en las campañas masivas gratuitas, y explorar estrategias para incrementar la participación en futuros esfuerzos.
Metodología/Principales Hallazgos
Realizamos 8 grupos focales (GF) en comunidades urbanas y peri-urbanas del distrito de Mariano Melgar; cada uno incluyó personas de ambos sexos, y participantes y no participantes en las campañas. Todos los GF fueron transcritos, codificados independientemente por duplicado, y los resultados fueron resumidos usando el Modelo Social Ecológico. A nivel individual, los participantes describieron no conocer suficiente sobre la rabia y sobre las campañas de vacunación, desconfianza sobre la campaña, e incapacidad para manejar a sus perros; esto fue mayor en el área peri-urbana. A nivel interpersonal, detectamos presión social para vacunar a los perros, pero también reportes de burlas y mofa hacia aquellos que invierten tiempo y dinero en cuidar a sus perros. A nivel organizacional, los participantes consideraron que la información recibida acerca de la campaña era insuficiente y llegaba a destiempo, que la localización de los puestos de vacunación era inadecuada, y que el personal de la campaña era muy limitado. A nivel de la comunidad, la influencia del paisaje y la topografía en la accesibilidad a los puntos de vacunación fue reportada de manera distinta en las áreas urbanas y peri-urbanas. La baja seguridad ciudadana y los pobres materiales de construcción en las áreas peri-urbanas también resultan en un mayor número de perros guardianes; estos perros usualmente están sueltos en la calle y son más difíciles de manejar y llevar a los puestos de vacunación.
Conclusiones
Una campaña de comunicación podría incrementar el conocimiento sobre la rabia canina. Mensajes anticipados y oportunos sobre dónde y cuándo la vacunación va a ocurrir podrían incrementar la autoeficacia de los dueños de los perros. Pequeños cambios en la implementación de las campañas en los puntos de vacunación podrían incrementar la confianza y motivación del público. La localización de los puestos de vacunación debería tomar en cuenta el paisaje y otras consideraciones a nivel de la comunidad.

Palabras clave: Canino; grupo focal; investigación cualitativa; peri-urbano; perro; rabia; vacunación masiva; zoonosis.
